# IL-10 expression defines an immunosuppressive dendritic cell population induced by antitumor therapeutic vaccination

**DOI:** 10.18632/oncotarget.13736

**Published:** 2016-12-01

**Authors:** Diana Llopiz, Marta Ruiz, Stefany Infante, Lorea Villanueva, Leyre Silva, Sandra Hervas-Stubbs, Diego Alignani, Elizabeth Guruceaga, Juan J Lasarte, Pablo Sarobe

**Affiliations:** ^1^ Program of Immunology and Immunotherapy, Center for Applied Medical Research (CIMA), University of Navarra, Pamplona, Spain; ^2^ IdiSNA, Instituto de Investigación Sanitaria de Navarra, Pamplona, Spain; ^3^ Cytometry Unit, Center for Applied Medical Research (CIMA), University of Navarra, Pamplona, Spain; ^4^ Bioinformatics Unit, Center for Applied Medical Research (CIMA), University of Navarra, Pamplona, Spain

**Keywords:** dendritic cells, therapeutic vaccination, IL-10, PD-L1, immunoregulation

## Abstract

Vaccination induces immunostimulatory signals that are often accompanied by regulatory mechanisms such as IL-10, which control T-cell activation and inhibit vaccine-dependent antitumor therapeutic effect. Here we characterized IL-10-producing cells in different tumor models treated with therapeutic vaccines. Although several cell subsets produced IL-10 irrespective of treatment, an early vaccine-dependent induction of IL-10 was detected in dendritic cells (DC). IL-10 production defined a DC population characterized by a poorly mature phenotype, lower expression of T-cell stimulating molecules and upregulation of PD-L1. These IL-10^+^ DC showed impaired *in vitro* T-cell stimulatory capacity, which was rescued by incubation with IL-10R and PD-L1-inhibiting antibodies. *In vivo* IL-10 blockade during vaccination decreased the proportion of IL-10^+^ DC and improved their maturation, without modifying PD-L1 expression. Similarly, PD-L1 blockade did not affect IL- 10 expression. Interestingly, vaccination combined with simultaneous blockade of IL-10 and PD-L1 induced stronger immune responses, resulting in a higher therapeutic efficacy in tumor-bearing mice. These results show that vaccine-induced immunoregulatory IL- 10^+^ DC impair priming of antitumor immunity, suggesting that therapeutic vaccination protocols may benefit from combined targeting of inhibitory molecules expressed by this DC subset.

## INTRODUCTION

The tumor microenvironment is characterized by the presence of immunosuppressive molecules which induce inhibitory effects on antitumor immunity [1]. This microenvironment precludes correct activation of antigen-presenting cells (APC) responsible for priming T-cell responses [2] and the effector phase of tumor-specific lymphocytes [3, 4]. Characterization of these immunosuppressive molecules has allowed the design of new therapies aimed at blocking their inhibitory functions, leading to activation of antitumor immunity and efficient clinical effects [5, 6]. Moreover, besides expression induced as a consequence of tumor growth, some therapies with an immunological component may also induce inhibitory elements as a negative feed-back mechanism [7]. Among them, vaccination induces not only T-cell-activating molecules, but also immunomodulatory mechanisms which regulate immune response activation [8–12].

IL-10 is a cytokine traditionally considered immunosuppressive due to its anti-inflammatory properties, mainly acting on APC by inhibiting expression of inflammatory cytokines and surface molecules associated with T-cell activation [13]. Different APC, including dendritic cells (DC), monocytes, macrophages and B-cells, as well as effector and regulatory T lymphocytes, produce IL- 10 [14]. However, besides inhibitory effects on APC with the concomitant down-regulation of T-cell activation, IL-10 may also have a stimulatory role by enhancing effector functions on activated CD8 T-cells and by activating NK cells through inhibition of MHC molecules [15–17]. In cancer patients, elevated IL-10 levels are associated with a poorer prognostic [18–20], suggesting that its inhibition would have a beneficial effect [21]. We and others have shown that inhibition of IL-10 during vaccination enhances the magnitude of T-cell responses [22, 23]. Furthermore, during therapeutic vaccination of mice bearing IL-10-expressing tumors, we have recently reported the relevance of vaccine-induced IL-10, as opposed to that derived from the tumor, demonstrating its importance in the control of T-cell activation and in the therapeutic efficacy of vaccines [23]. Therefore, characterization of events associated with IL-10 production during vaccination may allow the design of better antitumor therapies. In the present work we identify cell populations producing IL-10 during therapeutic vaccination, analyzing their properties and potential association with other immunosuppressive pathways. We have observed that IL-10 is induced in DC with a less mature phenotype and decreased T-cell activation capacity. Moreover, they express additional immunomodulatory molecules like PD-L1, which contribute to their poor immunogenicity. Characterization of these molecules in DC sets the rationale to design new strategies combining therapeutic vaccines with blockade of relevant associated immunosuppressive molecules.

## RESULTS

### DC consistently produce IL-10 in an Imiquimod-dependent manner

**Figure 1:**
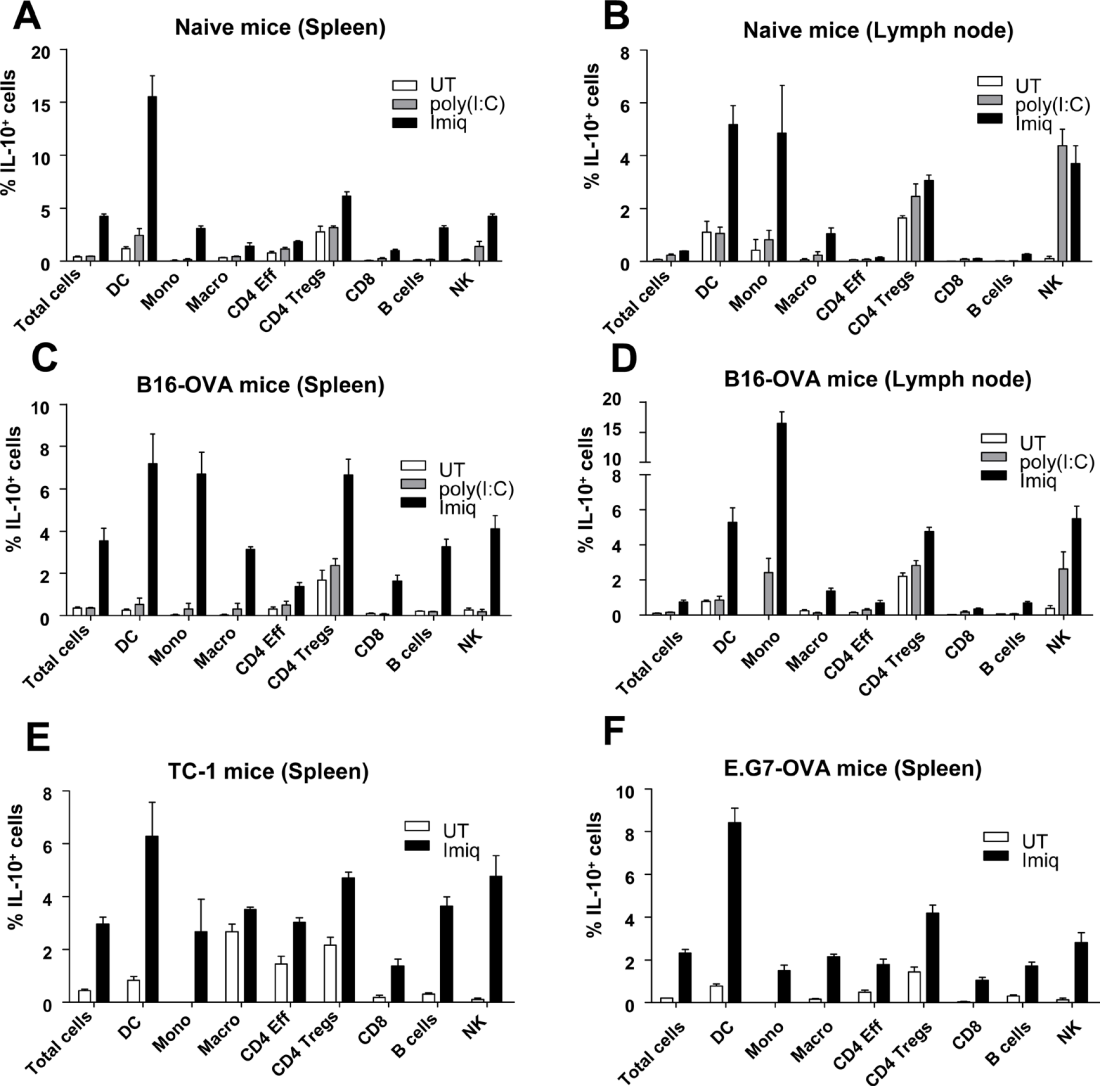
Identification of cell subsets producing IL-10 after therapeutic vaccination Naive Vert-X mice (**A–B**), mice bearing 5 mm B16-OVA (**C–D**), TC-1 (**E**) or E.G7-OVA tumors (**F**) (*n* = 8–11/group) were vaccinated with antigen (OVA in A–D and F or EDA-HPV-E7 in E) plus Imiquimod, antigen plus poly(I:C) or left untreated (UT). Two days later spleens or lymph nodes were obtained and the percentage of IL-10-producing cells was determined by flow cytometry in total cells and in the different subsets. Results correspond to the sum of 2–3 independent experiments.

To characterize relevant IL-10-producing cells induced during therapeutic vaccination, we used IL-10 reporter Vert-X mice [24]. Since IL-10 plays an inhibitory role even during vaccination of naive tumor-free mice [23], we started identifying IL-10^+^ cells in this setting. Mice were vaccinated with OVA+Imiquimod and compared to controls groups of untreated mice (UT) or mice vaccinated with OVA+poly(I:C), a vaccine in which IL-10 blockade did not provide any antitumor benefit [23]. In Imiquimod-vaccinated mice, contrary to those in UT and poly(I:C) groups, a higher proportion of total splenic IL-10^+^ cells was detected two days after treatment (Figure 1A). Most populations specifically produced IL-10 after Imiquimod vaccination (Figure 1A and Supplementary Figure S1). However, DC clearly surpassed values observed for other cells. Regarding lymph nodes, DC and monocytes showed the highest proportions of Imiquimod-specific IL-10^+^ cells (Figure 1B). Vaccination of mice bearing B16-OVA tumors showed that Imiquimod induced again a general increase in the total splenic population, corresponding to higher values in all subsets (Figure 1C), a result corroborated when analyzing tumor-draining lymph nodes (Figure 1D). As in tumor-free mice, DC and monocytes showed the highest proportion of IL-10^+^ cells. High values were also observed in Tregs and NK cells, although this was not induced only by Imiquimod, since poly(I:C) also induced IL-10. Analysis of IL-10^+^ cells inside the tumor showed that most populations produced IL-10. However, IL-10 production was not Imiquimod-specific, since clear IL-10 production was detected in poly(I:C)-treated mice and even in some subsets in UT mice (as observed for Tregs), as we previously reported in tumor homogenates [23]. Of note, DC did not produce IL-10 in UT mice, and in vaccinated mice, probably due to adjuvant-induced migration, DC could not be detected in sufficient numbers to be analyzed (Supplementary Figure S2).

Equivalent vaccination experiments in mice bearing TC-1 and E.G7-OVA tumors showed that although in most splenic cell populations the proportion of IL-10-producing cells increased after vaccination with Imiquimod, DC was the cell subset with the highest proportion of IL-10^+^ cells (Figures 1E–1F). These results show that several subsets, but mainly DC, consistently upregulate IL-10 production after vaccination in an Imiquimod-dependent manner.

### IL-10 with inhibitory effects on T-cell activation is induced at early time points after vaccination

**Figure 2:**
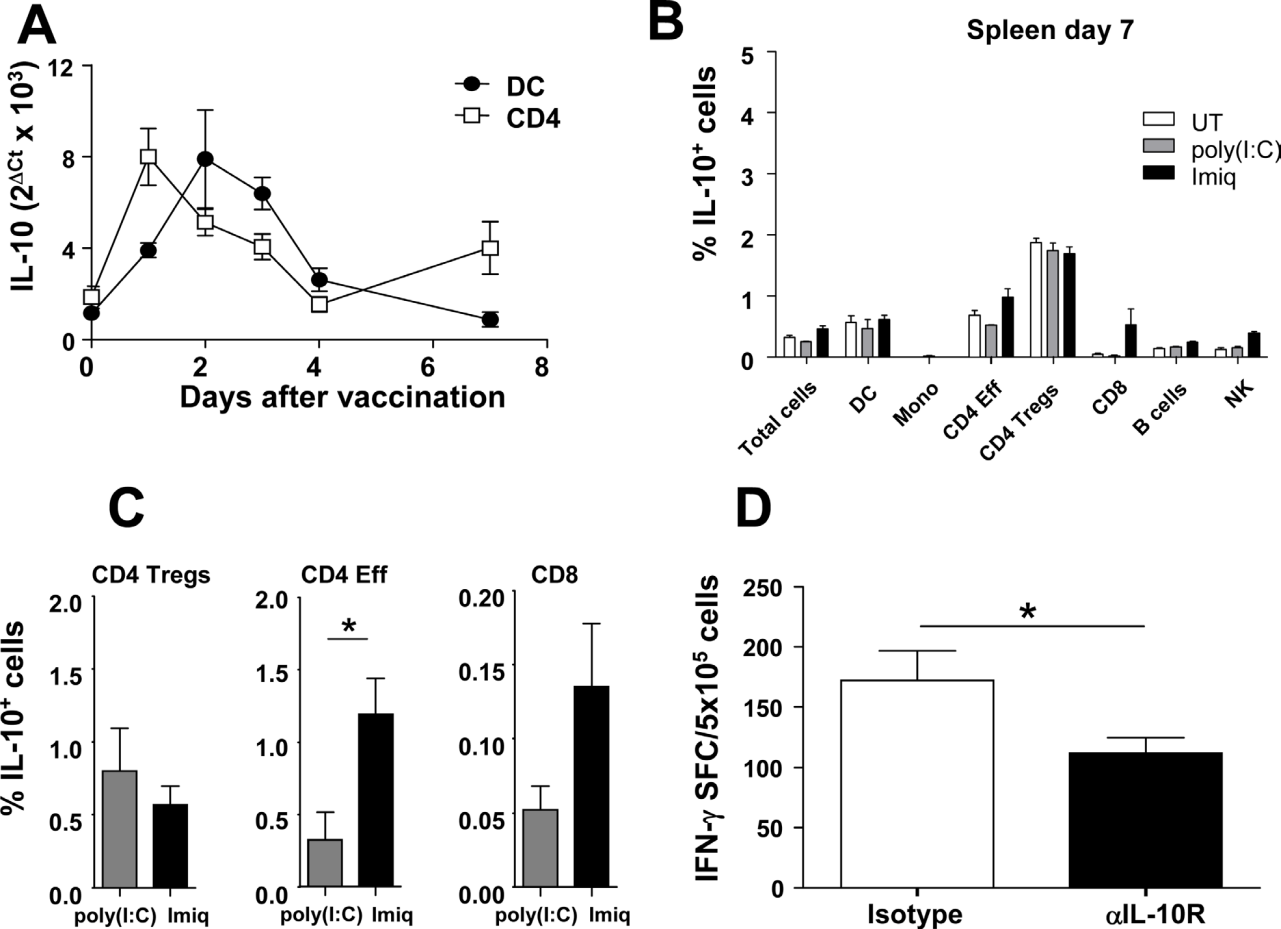
IL-10 with inhibitory effects on T-cell activation is induced at early time points after vaccination (**A**) C57BL/6 mice (*n* = 5/time-point) were vaccinated with OVA+Imiquimod and IL-10 mRNA was quantified by qPCR at different time-points in purified DC and CD4 cells. (**B**) Vert-X mice (*n* = 8/group) were vaccinated with OVA+Imiquimod, OVA+poly(I:C) or left untreated (UT) and one week later the percentage of splenic IL-10-producing cells was determined by flow cytometry. (**C**) C57BL/6 mice (*n* = 4) were vaccinated with OVA+Imiquimod or OVA+poly(I:C) and one week later splenocytes were stimulated with PMA/Ionomycin and intracellular IL-10 was determined by flow cytometry. (**D**) C57BL/6 mice (*n* = 4) were vaccinated with OVA+Imiquimod with or without blockade of IL-10 at day four after vaccination. At day 7, OVA-specific responses were determined by ELISPOT. Results are representative of 2 independent experiments.

To support that GFP expression observed in Vert-X mice indeed corresponded with IL-10, RT-PCR experiments measuring *Il10* mRNA were carried out in C57BL/6 mice vaccinated with OVA+Imiquimod. To avoid missing IL-10 production at time points other than day 2, time-course experiments were carried out from day 1 to 7. We analyzed *Il10* mRNA in purified splenic CD11c^+^ DC and CD4^+^ T-cells, representative of innate [25] and adaptive [22] cell populations producing IL-10. In DC IL-10 peaked at day 2, returning to basal levels at day 7, whereas in CD4^+^ T-cells, following a first peak at day 1 which decreased by day 4, a second, albeit weaker increase, was observed at day 7 (Figure 2A).

The second IL-10 peak observed at day 7 in CD4^+^ cells prompted us to study IL-10 production by other cell populations at this time point, using tumor-free mice, since equivalent results had been observed in lymphoid organs from tumor-free and tumor-bearing mice. Splenic CD4 Tregs maintained high Imiquimod-independent IL-10 production, whereas in remaining subsets a marginal Imiquimod-specific induction was observed only in effector CD4 and in CD8 and NK cells (Figure 2B), according to PCR results of CD4 cells shown in Figure 2A. Indeed, additional analyses of intracellular IL-10 using splenic cells from vaccinated C57BL/6 mice confirmed that effector CD4 and to a lesser extent CD8 T-cells, but not Tregs, specifically upregulated IL-10 in the Imiquimod group at day 7 (Figure 2C).

Since IL-10 blockade at day 0 enhanced T-cell responses [23], and two IL-10 peaks (an early peak mainly related to APC and a second peak related to T-cells) were detected, we studied the functional relevance of the second peak by blocking IL-10 at day 4 after vaccination. Blockade at this time-point did not provide any beneficial effect, indeed, poorer responses were obtained (Figure 2D), suggesting that the enhancement of immune responses observed after IL-10 blockade at day 0 is mainly due to inhibition of IL-10 produced at early time-points after immunization, during the priming phase.

### IL-10-producing DC have a different phenotypic and immunogenic profile and limit antitumor CD8 T-cell response activation

**Figure 3:**
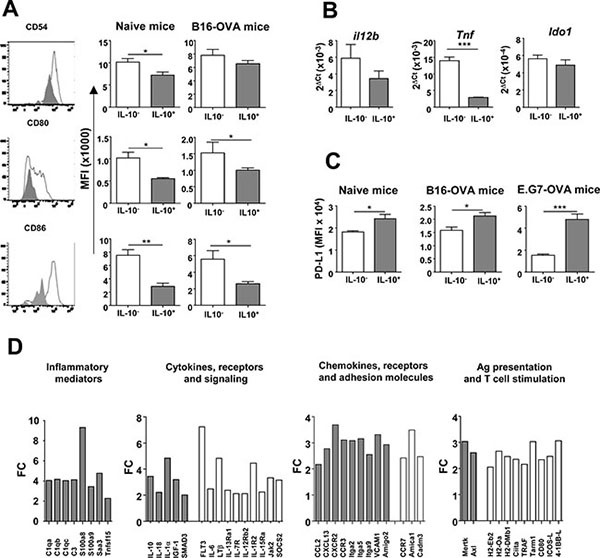
IL-10-producing DC have a different phenotypic and immunogenic profile (**A**) Maturation associated markers were determined in IL-10^−^ and IL-10^+^ DC from Vert-X mice (*n* = 4) with or without B16-OVA tumors two days after vaccination with OVA+Imiquimod. Left column corresponds to representative results of CD54, CD80 and CD86 molecules in DC from a vaccinated naive mouse, whereas middle and right columns show data corresponding to same markers in grouped naive and B16-OVA-vaccinated animals. (**B**) mRNA expression of several genes was analyzed by qPCR in purified IL-10^–^ and IL-10^+^ DC from Vert-X mice (*n* = 8) vaccinated as in A. (**C**) PD-L1 expression was measured by flow cytometry in DC from naive and B16-OVA and E.G7-OVA tumor-bearing mice (*n* = 4). Results are representative of 2–3 independent experiments. (**D**) Gene expression was analyzed in DC subsets. Results are shown as fold-change (FC), with upregulation in IL-10^+^ DC (gray bars) or in IL-10^−^ DC (white bars).

Due to the important proportion of IL-10^+^ APC induced by OVA+Imiquimod and considering the role that these cells play in T-cell priming (mainly for DC), phenotypic analyses comparing IL-10^+^ and IL-10^−^ cells were carried out after vaccination of naive and B16-OVA tumor-bearing mice. IL-10^+^ DC had a less mature phenotype than their IL-10^−^ counterparts, displaying a significantly lower expression of markers CD54, CD80 and CD86 associated to cell adhesion and T-cell co-stimulation (Figure 3A). However, in remaining APC populations, no differences were observed, except for higher CD80 values on IL-10^+^ B-cells (Supplementary Figure S3). This immature phenotype of IL-10^+^ DC was also observed in mice treated with other IL-10-inducing vaccines. Indeed, EDA-OVA+MAC, a multiple adjuvant combination [26] whose antitumor effects also increased after IL-10 blockade [23] or OVA+LPS, [27] also induced IL-10^+^ DC with a similar phenotype (Supplementary Figure S4).

Additional features of IL-10^+^ DC were studied in purified splenic DC from Imiquimod-vaccinated mice. Regarding stimulatory cytokines, qPCR experiments showed that IL-10^+^ DC had lower levels of transcripts for *Tnf* and *Il12b* (encoding TNF-α and IL-12 p40, respectively). Interestingly, in the case of regulatory molecules, although no differences were observed in the expression of the tryptophan-catabolizing enzyme *Ido1* (Figure 3B), higher surface PD-L1 expression was found in IL-10^+^ DC after immunization of naive mice or mice with different tumors (Figure 3C). These differences between IL-10^+^ and IL-10^−^ DC in activating and inhibitory molecules led us to carry out transcriptomic studies. Differentially expressed genes were grouped in Gene Ontology terms and most over-represented terms corresponded to regulation of immune system process (*p* = 2.13 × 10^−12^), leukocyte activation (*p* = 7.19 × 10^−9^), immune response (*p* = 1.08 × 10^−8^) and cell activation (*p* = 9.97 × 10^−8^). Analyses of individual genes showed that IL-10^+^ DC had a profile characterized by the expression of inflammatory mediators, cytokines, chemokines and receptors attracting inflammatory cells, while simultaneously displaying lower levels of molecules associated to antigen processing/presentation and T-cell co-stimulation (Figure 3D), suggesting that although these cells may be activated, they are not properly endowed for T-cell priming.

**Figure 4:**
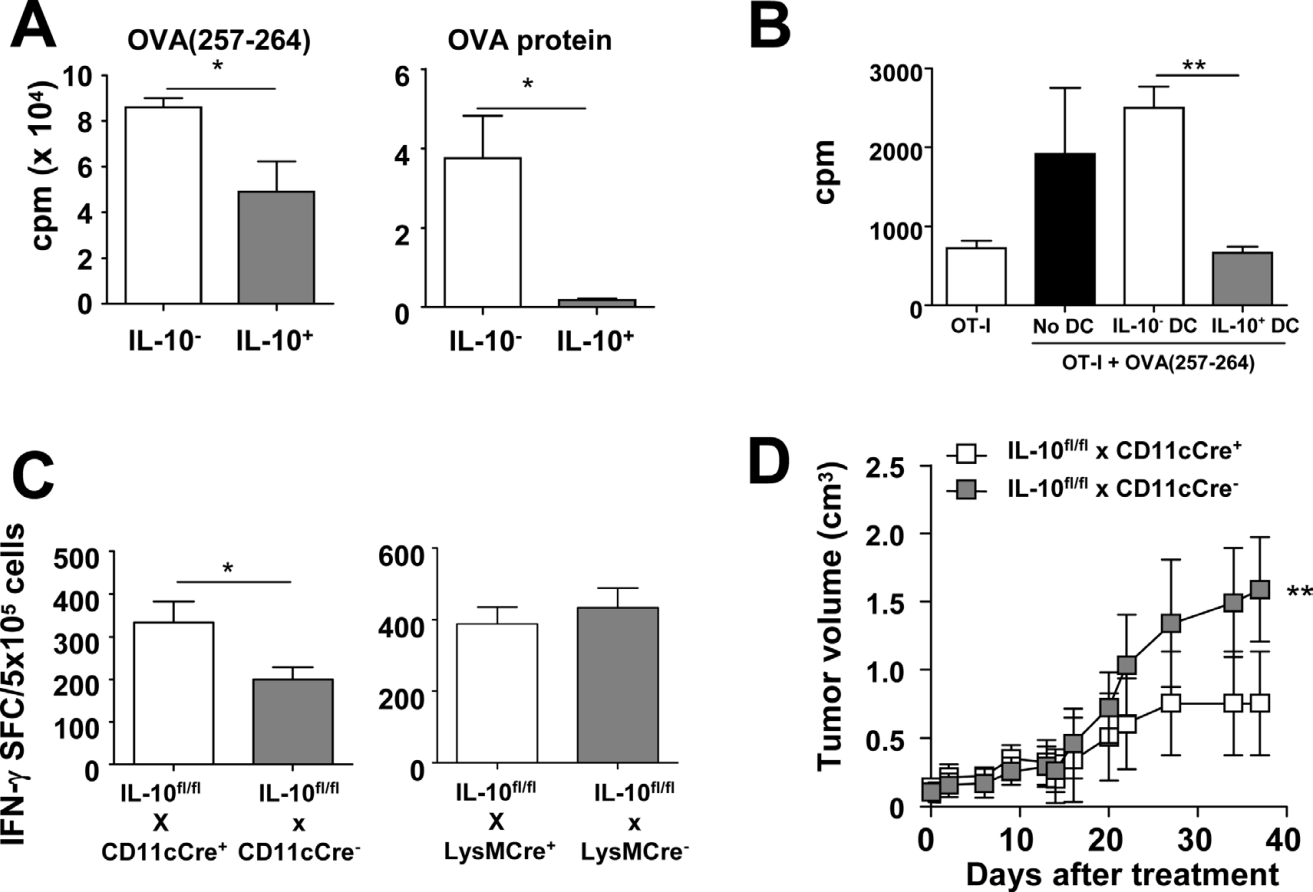
IL-10-producing DC have a poorer T-cell stimulatory capacity and limit the efficacy of anti-tumor therapeutic vaccination (**A**) Purified IL-10^−^ and IL-10^+^ DC from Vert-X mice (*n* = 8–10) were pulsed with peptide OVA(257–264) or OVA protein and used to stimulate OT-I CD8 T-cells. T-cell proliferation was determined three days later. (**B**) Total OT-I splenocytes (OT-I Spl) were pulsed or not with peptide OVA(257–264) and after washing the peptide, some wells additionally received IL-10^−^ or IL-10^+^ DC and T cell proliferation was measured as above. (**C**) IL-10^fl/fl^ x CD11cCre^+^ and IL-10^fl/fl^ x CD11cCre^−^ mice (*n* = 4–5/group) or IL-10^fl/fl^ x LysMCre^+^ and IL-10^fl/fl^ x LysMCre^−^ mice (*n* = 6/group) were immunized with OVA+Imiquimod and one week later OVA-specific responses were determined by ELISPOT. Results are representative of 2–3 independent experiments. (**D**) IL-10^fl/fl^ x CD11cCre^+^ and IL-10^fl/fl^ x CD11cCre^−^ mice (*n* = 6–8/group) bearing 5 mm E.G7-OVA tumors were treated with three weekly vaccination cycles with OVA + Imiquimod and tumor volume was monitored.

Therefore, we next studied the capacity of these DC subsets to activate T-cell responses. *In vitro* antigen presentation assays showed that IL-10^+^ DC induced lower proliferation of OT-I CD8 T-cells than IL-10^−^ DC. Interestingly, differences were clearer when using the whole OVA protein as antigen, compared with experiments using OVA(257–264) peptide, possibly related to defects in antigen processing/presentation machinery (Figure 4A). Most importantly, IL-10^+^ DC not only had a lower direct T-cell stimulatory capacity, but also actively inhibited T-cell stimulation induced by third party APC, reinforcing their immunosuppressive role (Figure 4B). To test *in vivo* the functional relevance of DC-derived IL-10, vaccination experiments were carried out in IL-10^fl/fl^ x CD11c-Cre^+^ mice, selectively lacking IL-10 in DC, or in IL-10^fl/fl^ x LysM-Cre^+^ mice, lacking IL-10 mainly in myeloid cells (monocytes/macrophages/granulocytes). Immunization with OVA+Imiquimod induced stronger T-cell responses in mice lacking IL-10 in DC than in their littermates with IL-10-proficient DC. However, no statistically significant differences were observed in mice lacking IL-10 in myeloid cells (Figure 4C). More importantly, similar to the results obtained when combining therapeutic vaccination plus systemic IL- 10 blockade [23], treatment of E.G7-OVA tumor-bearing mice with the OVA+Imiquimod vaccine had a stronger antitumor effect in mice lacking IL- 10 in DC than in those having competent DC (Figure 4D). Indeed, whereas only 16% of mice with IL-10^+^ DC rejected their tumor, 67.5% of tumors were rejected in mice lacking IL- 10 in DC. Overall, these results stress the importance of IL-10^+^ DC which, besides producing this immunosuppressive factor, are in a less-immunogenic condition, both at the phenotypical and functional level, leading thus to poorer CD8 T-cell priming during vaccination and restraining treatment efficacy.

### In vivo IL-10 blockade rescues mature phenotype of DC

**Figure 5:**
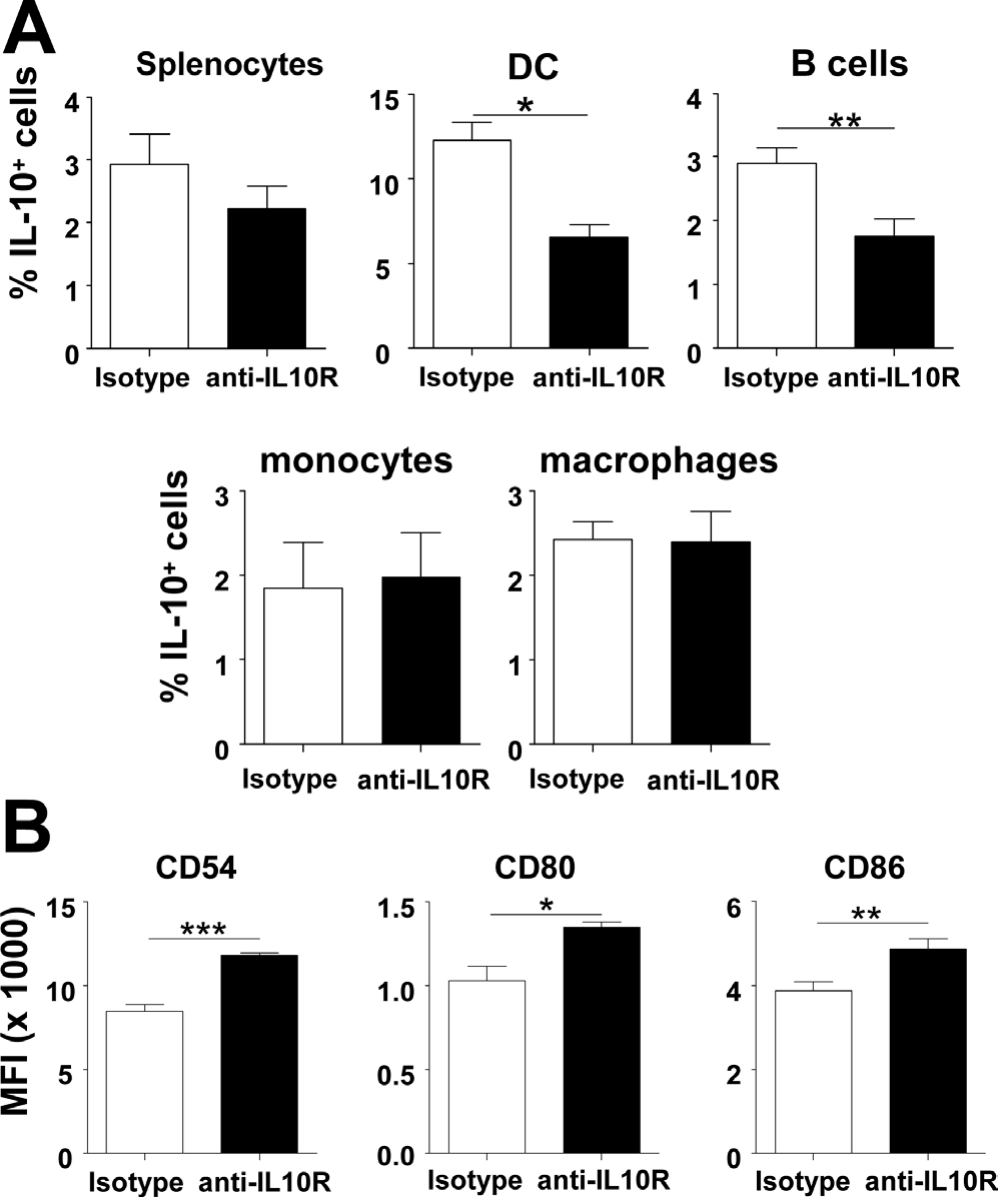
*In vivo* IL-10 blockade rescues mature phenotype of DC (**A**) Vert-X mice (*n* = 4/group) were vaccinated with OVA+Imiquimod with or without IL-10 blockade and 2 days later the proportion of total splenic cells and DC, B-cells, monocytes and macrophages producing IL-10 was determined by flow cytometry. (**B**) Phenotype of IL-10^+^ DC from vaccinated mice was also analyzed in these groups. Results are representative of 2–3 independent experiments.

We described that IL-10 blockade during therapeutic vaccination increased T-cell responses, associated with a more mature DC phenotype and enhanced functions, such as IL-12 production [23]. Thus, to dissect this rescuing effect of IL-10 blockade, IL-10-producing cells were analyzed in Vert-X mice vaccinated with OVA+Imiquimod, subjected or not to IL-10 blockade. A trend leading to a lower proportion of IL-10^+^ cells was observed in whole splenic cells after IL-10 blockade, which was statistically evident when specifically examining DC and B-cells. However, no differences were found in monocytes or macrophages (Figure 5A). Next, the effect of IL-10 blockade on DC phenotype was studied, according to their ability to produce IL-10. IL-10 blockade did not have any effect on IL-10^−^ DC phenotype (data not shown). Interestingly, increased expression levels of co-stimulatory molecules were found in IL-10^+^ DC from mice treated with blocking antibodies (Figure 5B). These results indicate that both mechanisms, lower proportion of IL-10^+^ DC and increased maturation, contribute to promote an overall higher number of mature DC after IL-10 blockade.

### Imiquimod-based vaccination induces DC with dual IL-10/PD-L1 expression whose simultaneous blockade increases antitumor efficacy of therapeutic vaccines

**Figure 6:**
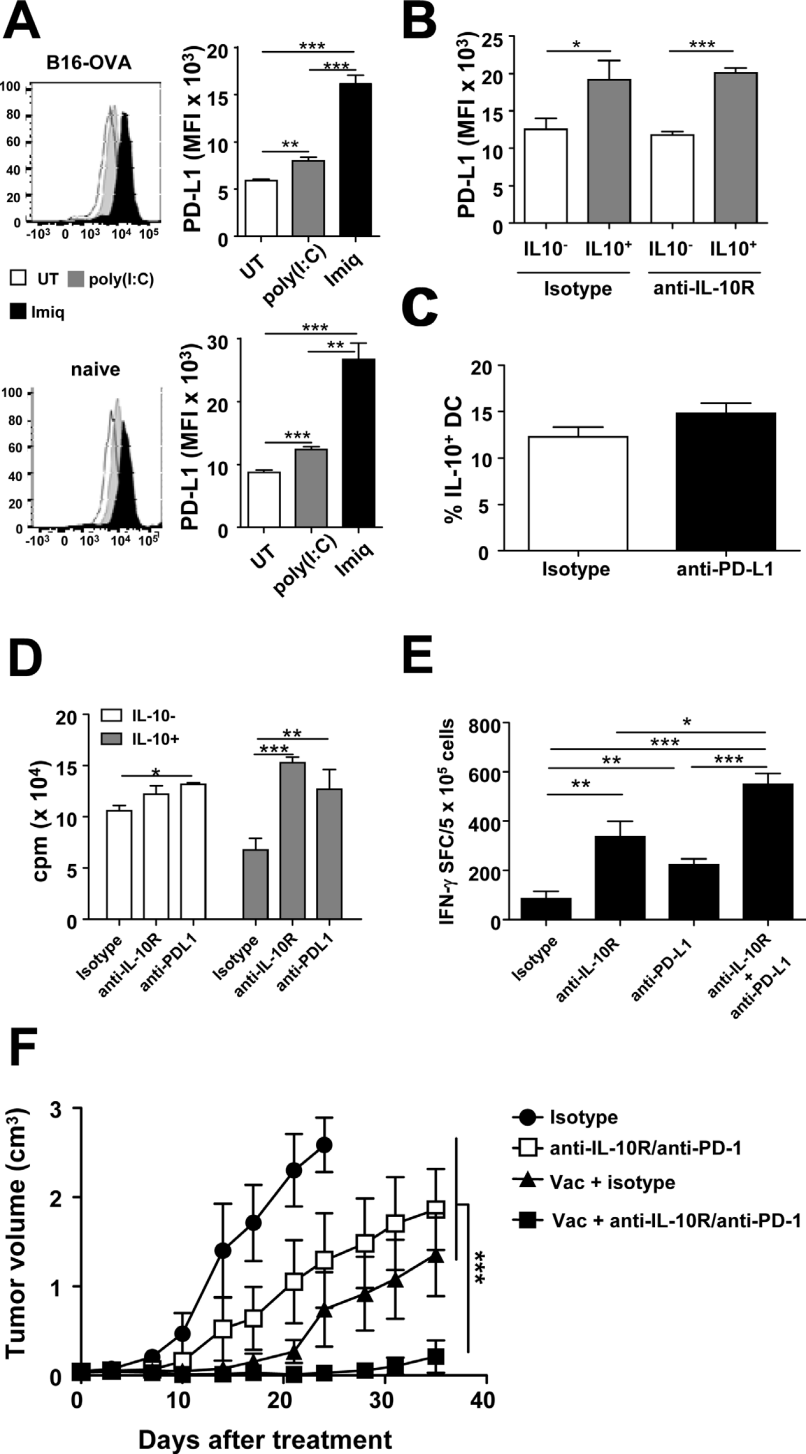
Blockade of vaccination-induced IL-10/PD-L1 in DC potentiates T-cell responses and increases antitumor therapeutic efficacy (**A**) Naive or B16-OVA tumor-bearing Vert-X mice (*n* = 4/group) were vaccinated with OVA+Imiquimod, OVA+poly(I:C) or left untreated. Two days later PD-L1 expression was determined in DC. (**B**) Vert-X mice (*n* = 4/group) were vaccinated with OVA+Imiquimod with or without IL-10 blockade and PD-L1 expression was determined two days later in IL-10^−^ and IL-10^+^ DC. (**C**) Vert-X mice (*n* = 4/group) were vaccinated with OVA+Imiquimod with or without PD-L1 blockade and the proportion of IL-10^+^ DC was determined two days later. (**D**) IL-10^−^ and IL-10^+^ DC obtained from OVA+Imiquimod-vaccinated mice were used to stimulate OT-I CD8 T-cells in the presence of OVA(257–264) plus control or IL-10R- or PD-L1-blocking antibodies. T-cell proliferation was determined three days later. (**E**) C57BL/6 mice (*n* = 4/group) were vaccinated with OVA+Imiquimod together with control or IL-10R- or PD-L1-blocking antibodies and one week later OVA(257–264)-specific responses were determined by ELISPOT. (**F**) C57BL/6 mice (*n* = 7–8/group) bearing 5 mm B16-OVA tumors were treated with three weekly vaccination cycles with OVA+Imiquimod with or without IL-10R/PD-1-blocking antibodies and tumor volume was monitored. Results are representative of 2–3 independent experiments.

Despite their less mature phenotype, IL-10^+^ DC displayed higher levels of PD-L1 (Figure 3C), a molecule associated with TLR-activated DC [9, 28–30] which is involved in tumor immunosuppression [31]. This PD-L1 up-regulation was also observed in other IL-10^+^ APC (Supplementary Figure S5). We were thus interested in studying the association between IL-10 production and PD-L1 expression in different vaccination models. Examining whole splenocytes in B16-OVA-tumor-bearing mice, both poly(I:C)- and Imiquimod-based vaccines enhanced the proportion of PD-L1 expressing cells. However, Imiquimod, besides inducing IL-10, led to the highest percentages of PD-L1^+^ cells, a result also observed after vaccination of naive tumor-free mice (Supplementary Figure S6). When specifically considering DC, although most cells (> 95%) were PD-L1^+^ irrespective of the vaccine (data not shown), clear differences were observed when analyzing the intensity of PD-L1 expression. While the poly(I:C)-based vaccine induced some increase regarding the UT group, Imiquimod-vaccinated mice expressed the highest PD-L1 levels, after vaccination of both tumor-bearing and naive mice (Figure 6A).

Enhanced PD-L1 levels on IL-10^+^ cells and data concerning interactions in the expression of these molecules [32–34] prompted us to analyze a potential association in their mutual expression. Thus, Vert-X mice were vaccinated with OVA+Imiquimod, with or without blockade of these molecules. IL-10 blockade did not modify PD-L1 expression, neither in IL-10^+^ nor in IL-10^−^ cells. This was observed when studying the whole splenic cell populations (Supplementary Figure S7A) and separately in DC (Figure 6B), B-cells, monocytes and macrophages (data not shown). Similarly, PD-L1 blockade did not modify the proportion of vaccine-induced IL-10^+^ cells (Figure 6C and Supplementary Figure S7B). To test the functional relevance of these immunosuppressive factors in IL-10^+^ DC, we repeated *in vitro* antigen presentation assays combined with blocking antibodies. While PD-L1 blockade only induced a minimal increase in T-cell stimulation by IL-10^−^ DC, both IL-10R and PD-L1 blockade clearly led to enhanced T-cell responses induced by IL-10^+^ DC (Figure 6D), suggesting that these immunosuppressive factors play important and independent roles as inhibitory molecules in IL-10^+^ DC in the vaccination setting. Although PD-1 expression was not detected in CD8 T cells in lymphoid organs of naive mice one week after vaccination (data not shown) presumably due to the low frequency of Ag-specific cells induced by the vaccine, PD-1 was observed in tumor bearing mice, whose OVA(257–264) Tetramer^+^ frequencies ranged between 5 and 8%. Indeed, 20% of these Tetramer^+^ cells expressed PD-1 in tumor-draining lymph nodes whereas these values increased to 80% in tumor infiltrating T lymphocytes (Supplementary Figure S8), supporting that *in vivo*, similarly to IL-10 blockade, inhibition of PD-1/PD-L1 axis would enhance vaccine-induced antitumor T cell responses. Accordingly, the rescuing effect observed after *in vitro* blockade was translated *in vivo* in vaccination experiments with dual blockade. Both IL-10R and PD-L1 blocking antibodies enhanced vaccine-induced T-cell responses, which were clearly increased after combined administration (Figure 6E).

Since the combination of the vaccine with the simultaneous blockade of IL-10R and PD-L1 gave the best results in immunization experiments, we tested the antitumor therapeutic relevance of this strategy in mice bearing B16-OVA tumors. We chose anti-PD-1 antibodies to inhibit the PD-1/PD-L1 pathway, similarly to initially approved therapies against this axis. Although individual therapies based on vaccination or inhibitory antibodies had some effect on tumor growth, combination therapy potently inhibited tumor growth (Figure 6F).

## DISCUSSION

Activation of immunity is accompanied by concomitant triggering of self-regulatory mechanisms to avoid excessive responses with harmful effects. However, in cancer, either during tumor progression or as a consequence of therapies, some control mechanisms are over-expressed and preclude correct induction of antitumor responses. Here we have analyzed the role of IL-10, a cytokine with controversial effects on tumor immunity [16], reported as detrimental for T-cell priming during therapeutic vaccination [23, 25]. We have observed in different tumor models that, although several cell subsets may produce IL-10 upon Imiquimod-based vaccination, APC (namely DC) consistently produce IL-10 at early time-points. Tregs or NK cells may also produce important amounts of IL-10; however, this source does not seem to be relevant, since it occurs in the absence of vaccination or with vaccines where IL-10 inhibition is not beneficial. Previous studies in Imiquimod-vaccinated mice have shown the importance of IL-10. Although the relevant IL-10 source was not identified, Tregs and B-cells were discarded [35], in agreement with our results highlighting DC. Immunomodulatory IL-10 produced by DC at early time points [25] is congruent with the role these cells play at initial stages of T-cell activation, whereas production at later time points (e.g., day 7) is associated with Tr1-derived IL-10, as reported in a prolonged Imiquimod-based therapy model [22]. Several issues stress the relevance of IL-10^+^ DC. First, there is a phenotypical and functional impairment specifically associated to DC expressing this cytokine. Indeed, IL-10 production is associated not only with a less mature DC phenotype (not observed in other APC subsets), but also with a functional impairment in T-cell priming, shown as a poorer stimulatory capacity and as a direct suppressive capacity. Second, the enhanced antitumor immunity resulting from IL-10 blockade during vaccination is associated with effects at the DC level, both by decreasing the number of IL-10^+^ DC and by upregulating maturation-associated molecules in these cells. Finally, selective depletion of DC-derived IL-10, but not in other APC, leads to improved *in vivo* responses and antitumor effect. It is interesting to note that not all adjuvants used have shown the same capacity to *in vivo* induce IL-10-producing DC. Indeed, whereas these cells were not detected in mice immunized with poly(I:C)-based vaccines, they were clearly observed when using Imiquimod, LPS or a multiple adjuvant combination (MAC). We previously reported the capacity of MyD88-depending adjuvants (Imiquimod, CpG oligonucleotides) to induce *in vitro* IL-10 production by DC, as opposed to those independent on MyD88 (poly(I:C), anti-CD40 agonistic antibodies) [23]. Interestingly, it has been reported that IL-10 produced by DC dampens MyD88-dependent, but not MyD88-independent signaling [36], suggesting that signalling through receptors which depend on this pathway may be associated to the inhibitory effect that we observe in DC.

Immunoregulatory IL-10^+^ DC have been recently described in murine models of sustained inflammation and immunosuppression like chronic infections, characterized by their poor stimulatory capacity associated with increased expression of negative regulatory factors [37, 38]. However, as opposed to IL-10^+^ DC found in our vaccination setting, those IL-10^+^ DC expressed higher CD80 and CD86 levels [37]. Vaccine-associated acute inflammation versus chronic inflammation corresponding to those settings might explain these differences. Interestingly, in humans, individuals treated with Imiquimod show an early peak of IL-10 at day 2 [39]. Moreover, human DC grown in an IL-10 rich environment display an immature phenotype together with increased IL-10 and PD-L1 expression [40], resembling our results.

Among immunoregulatory molecules over-expressed in IL-10^+^ DC, PD-L1 is of special interest due to its prominent role in immunoregulation and the availability of new clinical treatments targeting this pathway [41, 42]. Although DC activation leads to PD- L1 upregulation [9, 28–30], our results show that not all vaccine adjuvants promote PD-L1 expression to the same levels. Thus, whereas poly(I:C) upregulates PD- L1 to some extent, Imiquimod induces the highest PD-L1 levels, more evidently in IL-10^+^ DC, reinforcing the immunoregulatory role of this subset. PD-L1 and IL- 10 have been shown to inhibit anti-tumor responses in different tumors [18–20, 43]. Although their expression can be mutually regulated [32–34], in our vaccination setting IL-10 and PD-L1 do not exert reciprocal effects. Prolonged expression of these molecules in established tumors, contrary to the short-time induction we see during vaccination, may explain discrepancies observed in the regulation of their mutual expression, resulting in different pathways that induce and control their presence. Interestingly, this expression confers upon them separate inhibitory mechanisms and their blockade independently rescues antigen-presenting properties of IL-10^+^ DC, suggesting that they could be considered as separate targets in strategies aimed at enhancing T-cell responses by potentiating DC activity. Accordingly, their combined blockade during vaccination induces stronger antitumor responses, resulting in a higher therapeutic efficacy. PD-1/PD-L1 blocking therapies are currently being used with interesting results preferentially in PD-L1^+^ tumors. However, besides tumor cells, infiltrating immune cells may also express inhibitory PD-L1 [43]. Our results add a new element to this setting, suggesting a superior beneficial effect of those combination therapies which include vaccines and these checkpoint inhibitors. It has been recently reported that in melanoma patients, NY-ESO-1-specific CD8 T-cells expressing high PD-1 levels upregulate IL-10R upon antigen recognition, being inhibited thus by these two immunosuppressive pathways [44]. Interestingly, simultaneous blockade of these targets promoted expansion and antitumor functions of these cells, resembling our *in vivo* vaccination results. These data suggest that Imiquimod-based vaccination clinical trials [45] could benefit from simultaneous blockade of IL-10 and PD1/PD-L1.

Finally, the concept of antitumor therapeutic vaccine is currently being expanded from the classical antigen+adjuvant administration to therapies that release tumor antigens associated to immunogenic cell death [46]. Some endogenous alarm molecules induced by conventional therapies (e.g. HMGB1) signal in DC through TLR4 [47], a pathway known to induce IL- 10 [27]. Thus, it is tempting to speculate that some DC stimulated through this pathway would resemble and acquire the properties of immunoregulatory DC described in the present work, suggesting that ongoing clinical trials combining immunogenic cell death-inducing agents, such as doxorubicin, with anti-PD-1 antibodies (e.g. NCT02181738, NCT02499367, NCT02622074, NCT02331251; clinicaltrials.gov ) might also benefit from IL-10 blockade.

In summary, we have described an IL-10^+^ DC subset induced by vaccination, with an immature phenotype and poor stimulatory capacity, associated with inhibitory molecules such as PD-L1. Combination of vaccines with blockade of these or additional immunosuppressive targets expressed by these cells may yield more efficient immunotherapeutic protocols.

## MATERIALS AND METHODS

### Antigens

Peptide OVA(257-264) > 95% pure (NeoMPS) and immunogens OVA (low endotoxin; Hyglos) and EDA-HPVE7 have been previously described [23].

### Mice

Vert-X (B6(Cg)-Il10^tm1.1Karp^/J), OT-I (C57BL/6-Tg(TcraTcrb)1100Mjb/J) and LysMCre (B6.129P2-*Lyz2^tm1(cre)Ifo^*/J) mice were obtained from Jackson. IL- 10^fl/ fl^ (Il10^tm1Roer^) and CD11cCre (Tg(Itgax-cre)1-1Reiz) mice were kind gifts from Drs. A. Roers (Institute for Immunology, Dresden; Germany) and D. Sancho (CNIC, Madrid; Spain), respectively. Female C57BL/6 were from Harlan (Barcelona, Spain). They were maintained in pathogen-free conditions and treated according to guidelines of our institution, after study approval by the review committee.

### Cell lines

B16-OVA tumor cells (obtained from Dr. G. Kroemer; Paris, France), E.G7-OVA (from ATCC) and TC-1-P3(A15) cells, (obtained from Dr. T.-C. Wu; Baltimore, USA) were grown as described [23]. Cell stocks were created upon cell line receipt and early passages were used for tumor experiments. They were routinely tested for mycoplasma. Re-authentification of cells was not performed since receipt.

### Immunization of mice

Mice were injected with B16-OVA cells (10^5^, intradermally), TC-1-P3(A15) (10^5^, subcutaneously) or E.G7-OVA (5 × 10^5^, subcutaneously) and when the tumor diameter reached 4–5 mm, they received intratumoral administration of OVA protein (0.5 mg/mouse) or EDA-HPVE7 immunogen [23] (2 nanomoles) combined with Imiquimod cream (Meda-Aldara™; topical application; 2.5 mg/mouse), poly(I:C) (Amersham; 50 μg/mouse; intratumor) or left untreated. Tumor-free mice received similar immunizations by subcutaneous route. At different time-points they were sacrificed and splenocytes, lymph node cells or tumor-infiltrating cells were obtained for characterization. Additionally, they received i.p. injection of anti-IL-10R (500 μg), anti-PD-L1 (200 μg) or the corresponding isotype control antibodies (all from BioXcell).

### Tumor treatment experiments

Mice bearing 4–5 mm B16-OVA or E.G7-OVA tumors received 3 weekly cycles of OVA (intratumor; 0.5 mg/mouse) combined with Imiquimod at day 0 as described [23]. Some groups received anti-IL-10R, anti-PD-1 or isotype antibodies as above at days 0, 7 and 14. Untreated mice bearing similar tumors were used as positive controls of tumor growth. Tumor volume was calculated using the formula: V= (length × width^2^)/2. Mice were killed when tumor diameter reached 17 mm.

### ELISPOT

T-cells producing IFN-γ were determined by ELISPOT (BD-Biosciences) as described [23]. Splenocytes (5 × 10^5^/well) were stimulated with peptide OVA(257–264) for 24 h and the number of spot-forming cells was enumerated with an automated counter.

### Flow cytometry

Lymphoid organs and tumors were obtained after vaccination, treated with collagenase and DNAse for 15 minutes and homogenized. Cells were incubated for 10 min with Fc Block™ (BD-Biosciences) and stained with specific antibodies. Analysis of cells producing IL-10 in Vert-X mice (GFP^+^ cells) was performed by using antibodies CD11c-BV570, F4/80-Pacific Blue, CD11b-APC, Ly6C-PE-Cy5 (all from Biolegend) and Ly6G-PE-Cy7 (BD-Biosciences), to define DC, monocytes and macrophages. CD3-PE-Cy5 (AbD serotec), NKp46-PE (BD-Biosciences), CD19-APC (BD-Biosciences), CD8 BV421 (Biolegend), CD4-BV570 (Biolegend) and CD25-PE-Cy7 (TONBO Biosciences) were used to define lymphocytic subsets (Supplementary Figure S1). For intracellular IL-10 detection in C57BL/6 mice, splenocytes were stimulated 4 h with PMA/Ionomycin in the presence of GolgiStop and GolgiPlug (BD-Biosciences), surface stained with CD3-PE-Cy5, CD8-BV421, CD4-BV570 and CD25-PE-Cy7 antibodies and intracellular stained with anti-IL10-PE (Biolegend). For APC characterization studies, splenocytes were stained in different panels combining CD11c-BV570, F4/80-BV421, CD11b-PE (BD-Biosciences), Ly6C-PE-Cy5, Ly6G-PE-Cy7, CD19-APC-Cy7 (Biolegend), CD80-BV421 (BD-Biosciences), CD54-APC (Biolegend), CD86-PE-Cy5 (Biolegend) or CD86-BV510 (Biolegend), I-A^b^-PE (BD-Biosciences) and PD-L1-PE (BD-Biosciences) antibodies. PD-1 expression on CD8 T cells was determined by staining cells obtained from spleen, lymph nodes or tumors with CD8-Pacific Blue (AbD Serotec), OVA(257–264)-Tetramer-APC (MBL) and PD-1-FITC (Miltenyi). Samples were acquired in a FACSCantoII flow cytometer (Becton Dickinson) and analyzed using Flowjo software (Tree Star Inc).

### Cell purification

For IL-10 RT-PCR time-course experiments in C57BL/6 mice, animals were vaccinated with OVA+Imiquimod and anti-CD11c-conjugated magnetic beads were used to purify splenic DC, whereas CD4 T-cells were purified by negative selection (Miltenyi; Germany). Splenic IL-10^+^ and IL-10^−^ DC from Vert-X mice vaccinated with OVA+Imiquimod used for PCR studies, microarrays and antigen presenting assays were purified by using anti-CD11c-conjugated magnetic beads followed by staining with CD11c-APC antibodies (BD-Biosciences) and separation with a FACSAria flow cytometer according to their expression of IL-10 (GFP).

### Real-time PCR

Purified DC were resuspended and RNA extracted (Simply RNA Kit; Promega). Real-time PCR was performed as described [26], using primers GGACAACA TACTGCTAACCG and AATCACTCTTCACCTGCTCC (IL-10), AGATGAAGGAGACAGAGGAG and GGAAA AAGCCAACCAAGCAG (IL-12 p40), CTTCCAGAACT CCAGGCGGT and GGTTTGCTCGACGTGGGC (TNFα), CCTTGAAGACCACCACATAG and AGCACCTTTCGAA CATCGTC (IDO) and CGCGTCCACCCGCGAG and CCTG GTGCCTAGGGCG (β-actin). Results were normalized according to b-actin. The amount of each transcript was expressed by the formula: 2^ΔCt^ (ΔCt = Ct(β-actin)-Ct(gene)).

### Microarrays

RNA samples from IL-10^+^ and IL-10^−^ DC were labeled, hybridized and scanned according to standard protocols (Affymetrix, Santa Clara, CA, USA). Briefly, 2 ng of RNA were processed using the Affymetrix GeneChip^®^ WT Pico Kit. Hybridization cocktails were hybridized to the GeneChip^®^ Mouse Gene 2.0 ST Arrays, incubated at 45°C for 16 hours, washed and stained on an GeneChip^®^ Fluidics 450 workstation (Affymetrix). They were scanned using the Affymetrix GeneChip^®^ Scanner 3000 7G. Data normalization was performed with RMA [48]. After quality assessment using R/Bioconductor [49], a filtering process was carried out to eliminate low expression probe sets. Applying the criterion of an expression value > 4 in both samples, 30436 probe sets were selected for analysis. Genes were selected as differentially expressed using a fold-change cut off > 1 and functional enrichment analysis of Gene Ontology categories was performed using the hypergeometric distribution in R. Data are publicly available in GEO database (accession number GSE84980).

### Proliferation assays

In direct antigen presentation assays purified DC (6 × 10^3^) and OT-I CD8 T-cells (1.8 × 10^4^) were co-cultured (*n* = 4 wells/condition) with or without peptide OVA(257–264) (1 μg/ml) or OVA protein (10 μg/ml). In some cases, antibodies against IL-10R, PD-L1 or isotype control (10 μg/ml) were also added. Two days later 0.5 μCi of H^3^-thymidine were added and cell proliferation was counted after overnight incubation. For suppression assays, 10^5^ OT-I total splenocytes were pulsed for 2 h with a suboptimal dose of OVA(257–264) (1 ng/ml), then peptide was extensively washed, 1.5 × 10^4^ unpulsed IL-10^+^ or IL-10^−^ DC were added and T cell proliferation was measured as above.

### Statistical analysis

Tumor growth was fitted to a third order polynomial and compared with the Extra sum-of-squares F test. Immune responses were analyzed using nonparametric Kruskal-Wallis and Mann-Whitney U tests. *p*<0.05 was taken to represent statistical significance.

## SUPPLEMENTARY MATERIALS FIGURES


