# Supplementary material for: IL-10 expression defines an immunosuppressive dendritic cell population induced by antitumor therapeutic vaccination
Source: Oncotarget. 2016 Dec 1;8(2):2659–71. doi: 10.18632/oncotarget.13736 (PMC5356831; doi:10.18632/oncotarget.13736)
Supplement: Supplementary file 1 [file oncotarget-08-2659-s001.pdf]

IL-10 expression defines an immunosuppressive dendritic cell population induced by antitumor therapeutic vaccination

Supplementary Materials

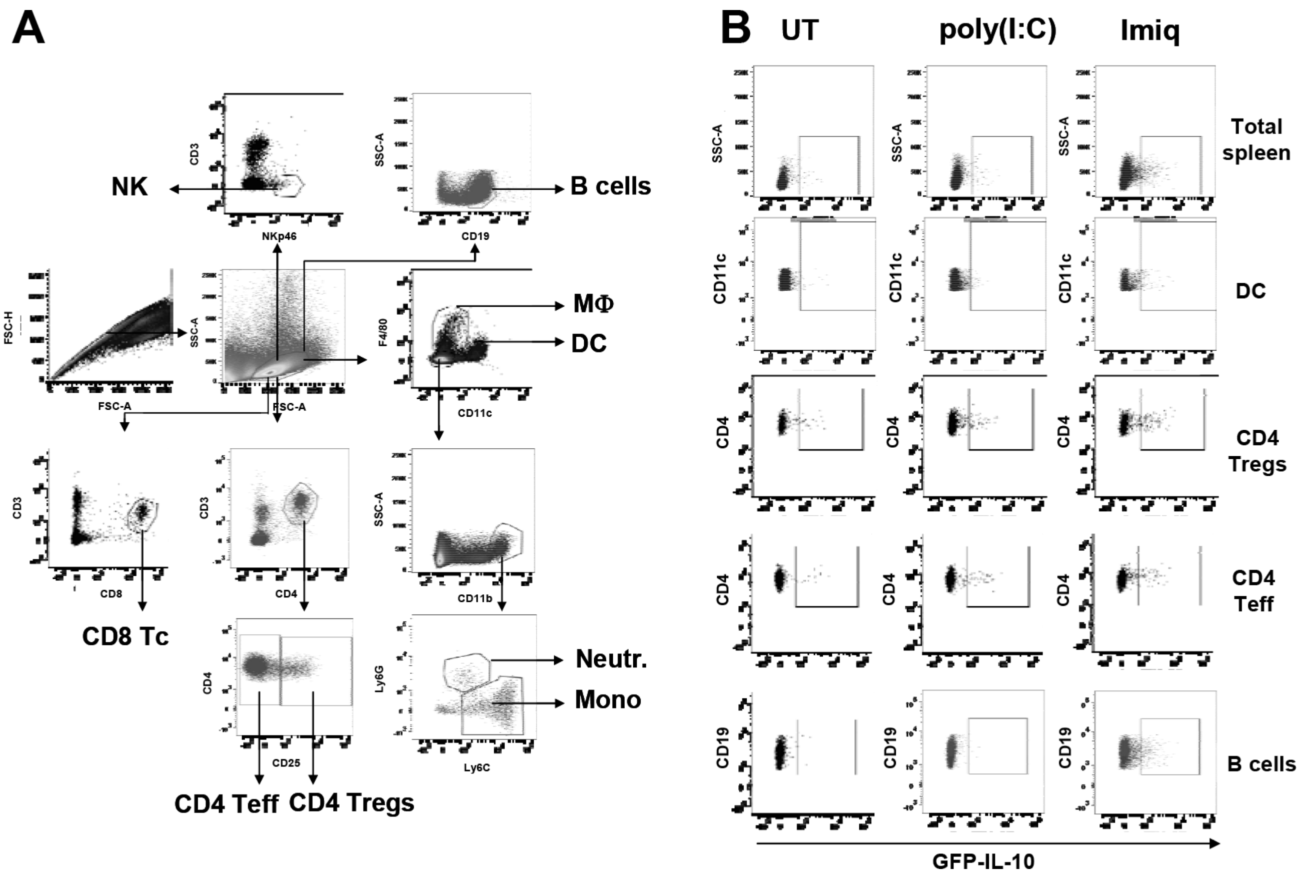

Supplementary Figure S1: Gating strategy to characterize IL-10-producing cell subsets in VertX mice and representative examples. Two days after treatment with vaccines combining OVA with poly(I:C), Imiquimod or left untreated (UT), VertX mice were sacrificed and organs were harvested and homogenized. (**A**) Cells were stained with a panel of antibodies against CD11c, F4/80, CD11b, Ly6C and Ly6G to define DC, macrophages (MF), monocytes and neutrophils, and a second panel with antibodies against NKp46, CD19, CD3, CD8, CD4 and CD25 to define NK cells, B cells, CD8 T cells and effector and regulatory CD4 T cells. (**B**) Characterization of IL-10-producing cells within total splenic cells and in representative cell subsets gated as described in A. Results correspond to analysis of splenic cells.

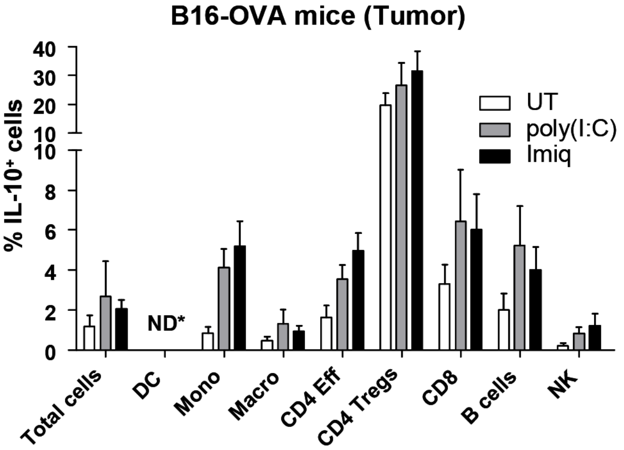

Supplementary Figure S2: Detection of intratumor IL-10-producing cells in VertX mice with B16-OVA tumors after different vaccination protocols. VertX mice bearing 5 mm B16-OVA tumors were vaccinated with OVA+Imiquimod, OVA+poly(I:C) or left untreated. Two days later they were sacrificed, tumors were harvested, homogenized and cells stained with panels of antibodies to define the different cell subsets. IL-10$^+$ cells were identified as GFP$^+$ inside each subset. IL-10$^+$ total cells and in different cell populations are shown. (*) ND: not detected in sufficient numbers to be analyzed in poly(I:C) and Imiq groups.

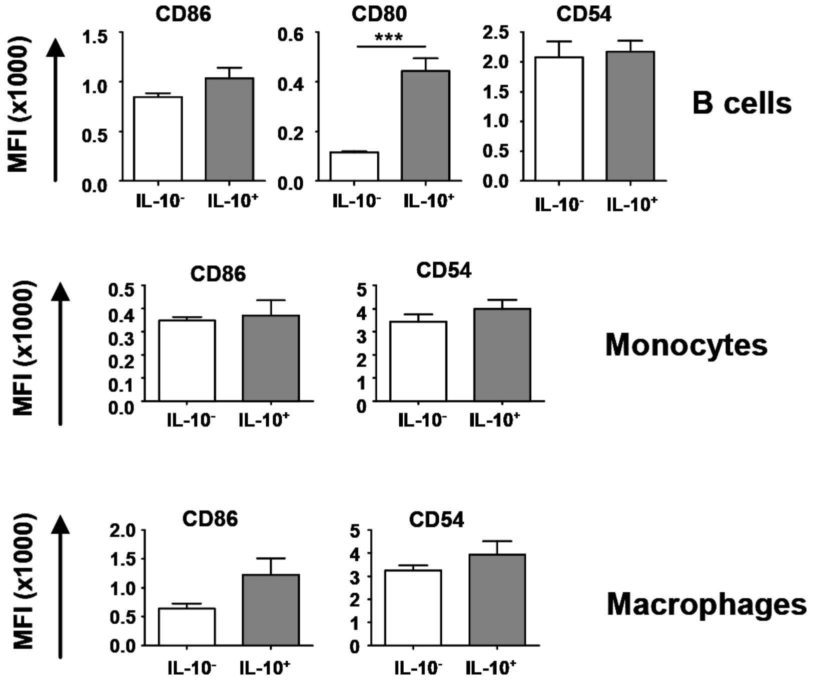

Supplementary Figure S3: Phenotypic characterization of IL-10$^+$ and IL-10$^-$ cells in non-professional APC subsets. VertX mice (n = 4/group) were immunized with OVA + Imiquimod and two days later spleens were harvested and the expression of maturation markers CD54, CD80 and CD86 was determined by flow cytometry in IL-10$^-$ and IL-10$^+$ B cells, monocytes and macrophages. Results are representative of two independent experiments.

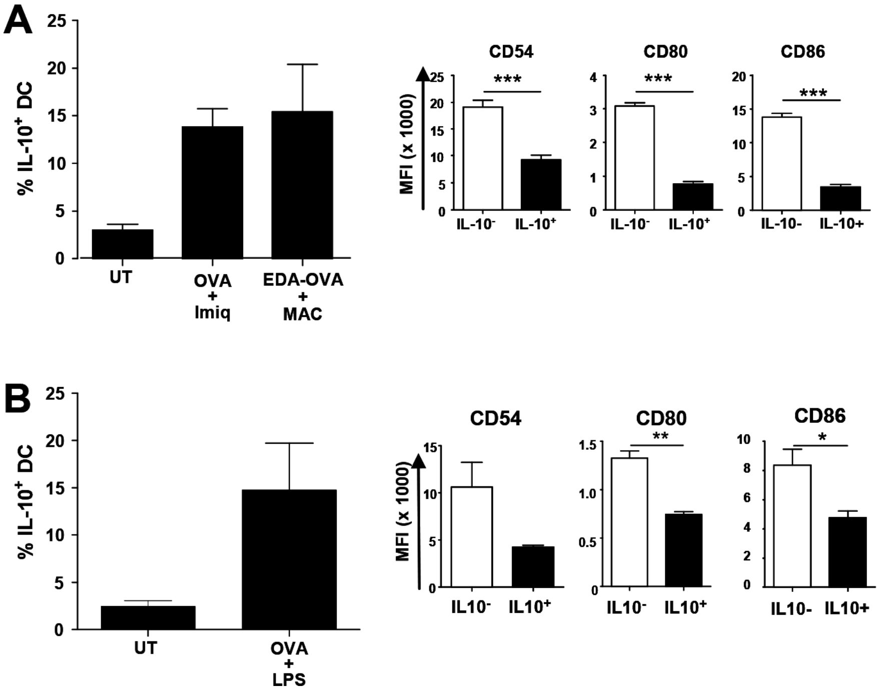

Supplementary Figure S4: IL-10$^+$ DC from mice immunized with different adjuvants display a less mature phenotype. VertX mice (*n* = 4/group) were immunized with (**A**) the multiple adjuvant combination (MAC) EDA-OVA + MAC (Aranda F et al, Cancer Res 2011) or with (**B**) OVA + LPS (30 µg/mouse). As control, mice were left untreated (UT) or immunized with OVA + Imiquimod. Two days later spleens were harvested and the percentage of IL-10$^+$ DC was determined as CD11c$^+$ GFP$^+$ cells. Expression of maturation markers CD54, CD80 and CD86 was determined by flow cytometry in IL-10- and IL-10+ DC from mice immunized with EDA-OVA + MAC (A) or OVA + LPS (B).

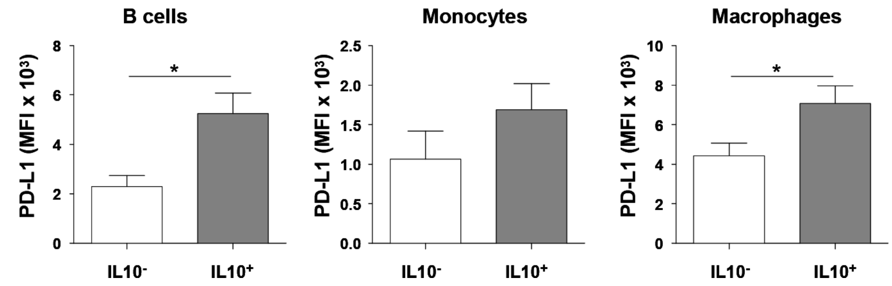

Supplementary Figure S5: Expression of PD-L1 in non-professional APC populations. VertX mice (*n* = 4/group) were immunized with OVA + Imiquimod and two days later spleens were harvested and expression of PD-L1 on IL-10$^-$ and IL-10$^+$ B cells (CD19$^+$), monocytes (CD11b$^+$, CD11c$^-$, Ly6C$^+$) and macrophages (F4/80$^+$) was determined by flow cytometry.

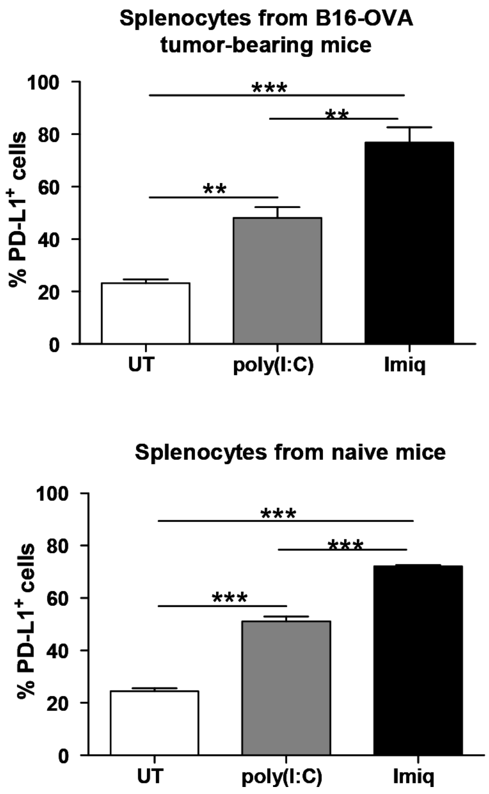

Supplementary Figure S6: Vaccination with Imiquimod enhances the proportion of PD-L1+ cells. VertX mice with or without 5 mm B16-OVA tumors (n = 4/group) were immunized with OVA + Imiquimod, OVA + poly(I:C) or left untreated. Two days later spleens were harvested and the proportion of PD-L1+ cells considering all splenocytes was determined by flow cytometry.

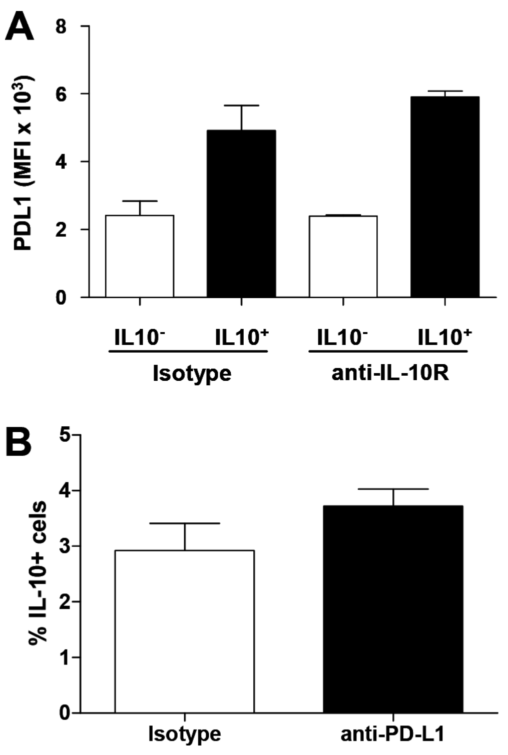

Supplementary Figure S7: Effect of IL-10 or PD-L1 blockade on the splenic expression of PD-L1 or IL-10, respectively. (**A**) VertX mice (n = 4/group) were immunized with OVA + Imiquimod plus anti-IL-10R or isotype control antibodies. Two days later spleens were harvested and the expression of PD-L1 on IL-10- and IL-10^{+} total splenocytes was analyzed by flow cytometry. (**B**) VertX mice (n = 4/group) were immunized with OVA + Imiquimod plus anti-PD-L1 or isotype control antibodies. Two days later spleens were harvested and the proportion of IL-10^{+} splenocytes was analyzed by flow cytometry.

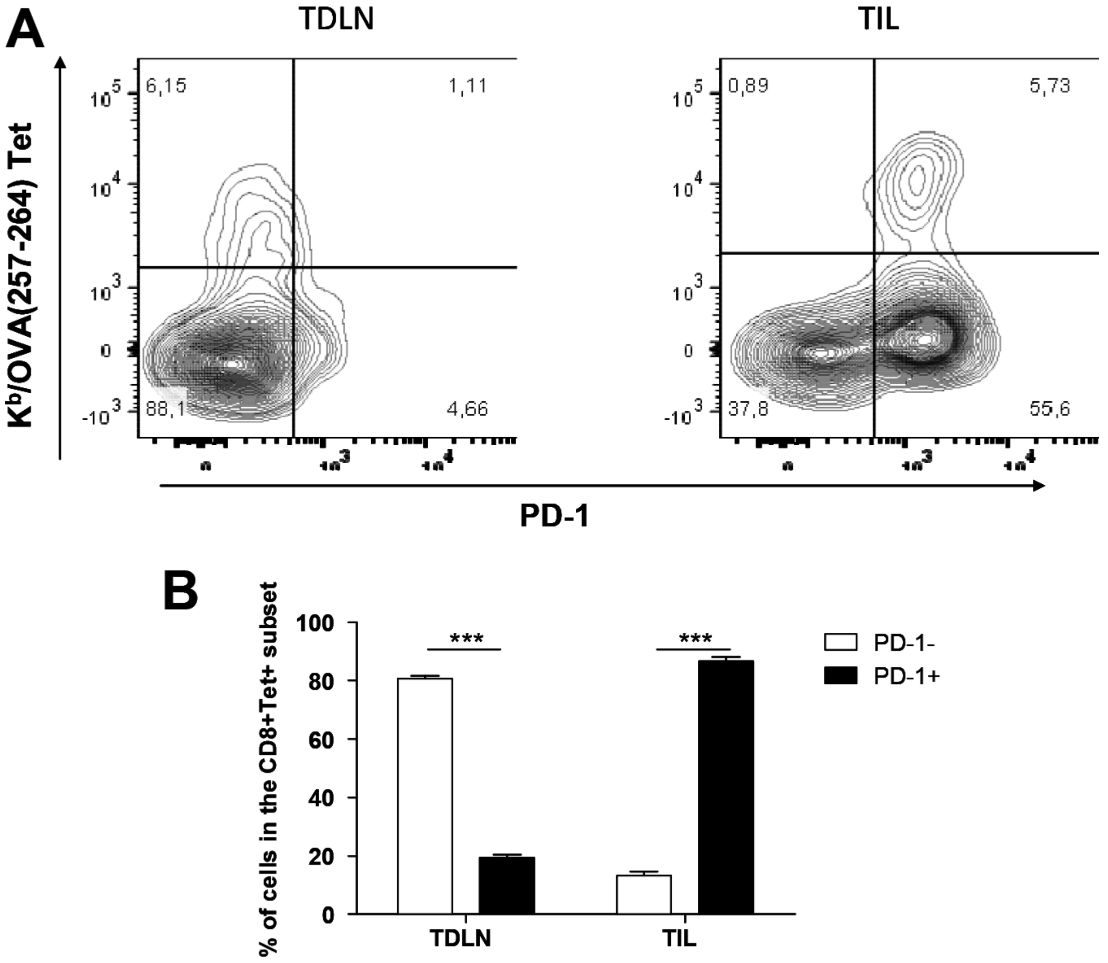

Supplementary Figure S8: PD-1 expression in tumor antigen-specific CD8 T cells in tumor-bearing mice. Cells were obtained from tumor-draining lymph nodes (TDLN) or tumors infiltrates (TIL) from C57Bl/6 mice (*n* = 3/experiment) bearing 8-10 mm B16-OVA tumors. They were stained with fluorochrome-labeled antibodies anti-CD3, -CD8, -PD-1 and Kb/OVA(257-264) tetramers. PD-1 expression was determined in CD8+ Tetramer+ cells. (**A**) Representative cases of CD8 cells in TDLN and TILs and (**B**) summary of an experiment out of two carried out.
